# Ex-vivo experimental strategies for assessing unconstrained shoulder biomechanics: a scoping review protocol

**DOI:** 10.12688/f1000research.72856.1

**Published:** 2022-01-21

**Authors:** Jeremy Genter, Eleonora Croci, Hannah Ewald, Andreas M. Müller, Annegret Mündermann, Daniel Baumgartner

**Affiliations:** 1 ZHAW School of Engineering, IMES Institute for Mechanical Systems, Winterthur, Switzerland; 2 Department of Biomedical Engineering, University of Basel, Basel, Switzerland; 3 Department of Orthopaedics and Traumatology, University Hospital Basel, Basel, Switzerland; 4 University Medical Library, University of Basel, Basel, Switzerland; 5 Department of Clinical Research, University of Basel, Basel, Switzerland

**Keywords:** Biomechanics, glenohumeral joint, human, muscle, simulator

## Abstract

**Background:** Shoulder biomechanics cannot be measured directly in living persons. While different glenohumeral joint simulators have been developed to investigate the role of the glenohumeral muscles in shoulder biomechanics, a standard for these simulators has not been defined. With this scoping review we want to describe available ex-vivo experimental strategies for assessing unconstrained shoulder biomechanics.

**Objective: **The scoping review aims at identifying methodological and/or experimental studies describing or involving ex-vivo simulators that assess unconstrained shoulder biomechanics and synthesizing their strengths and limitations.

**Inclusion criteria:** All unconstrained glenohumeral joint simulators published in connection with ex-vivo or mechanical simulation experiments will be included. Studies on glenohumeral simulators with active components to mimic the muscles will be included. We will exclude studies where the experiment is static or the motion is induced through an external guide, e.g., a robotic device.

**Methods:** We will perform database searching in PubMed, Embase via Elsevier and Web of Science. Two reviewers will independently assess full texts of selected abstracts. Direct backward and forward citation tracking on included articles will be conducted. We will narratively synthesize the results and derive recommendations for designing ex-vivo simulators for assessing unconstrained shoulder biomechanics.

## Introduction

The shoulder or glenohumeral joint is one of the most complex joints in the human body. The size of the glenoid fossa is much smaller than the articulating humeral head thereby facilitating a large range of motion but also making the joint prone to instability. Different tissues are present in the shoulder to provide more stability including, most importantly, the rotator cuff muscles. Furthermore, the glenohumeral capsule and some other muscles play a minor role in stabilizing the joint ^1^.

The glenohumeral joint and its stability has been studied in various conditions. Because joint load cannot be measured directly in the living person, previous studies have used *ex-vivo* approaches with shoulder simulators or in-silico methods such as musculoskeletal modelling approaches. Here, the focus on shoulder simulators aimed at investigating the passive biomechanics of the shoulder, such as joint stability due to joint reaction forces and its concavity ^2^, glenohumeral capsule stability ^3^ and overall stability of specific motions ^4^. Some research groups have also investigated the role of the muscles for glenohumeral biomechanics. To mimic the forces exerted by the muscles, various shoulder simulators have been developed.

Existing simulators usually consist of a clamping mechanism for the scapula and a cable pulley system that is attached to the tendons inserting at the humerus ^5– 7^. Although several simulators have been developed, to date there is no standard defining the design and technical requirements of such simulators. Depending on the specific research question, appropriate detailed simulators have been developed. In particular, shoulder simulators vary in three main aspects: the number of cables to mimic the investigated muscles; the degree of freedom (DOF) of the modelled joints; and the way the muscles are actuated. Existing simulators can be further categorized by the technical solution for generating muscle forces. The most trivial simulators have loaded the muscle cable pulley with passive loads such as springs or simply counterweights. More advanced simulators used active actuators such as pneumatic cylinders or motors to mimic the muscle forces. Although these simulators lack precision of the anatomical representation or physiological muscle recruitment, they are sufficient for answering many research questions and identifying new ones. Besides investigating solely the role of the muscles for shoulder biomechanics, these simulators are employed to address various research questions ranging from joint implants loading ^8^ to the effect of the rotator cuff muscle activation on glenohumeral kinematics ^7^ to the joint reaction forces during daily activity ^9^.

We performed a preliminary search in Pubmed and *JBI Evidence Synthesis,* with the search function of the journal's homepage, was conducted and only one systematic review ^10^ on the topic was identified. Williamson *et al.* ^10^ have conducted a systematic review on *ex-vivo* experiments for studying rotator cuff tear and instability. While they identified various experimental setups, only few of the included studies used active muscle forces. Furthermore, they categorized the *ex-vivo* experiments into three main topics: scapular orientation and mobility, muscle activation and humeral motion and condition of the glenohumeral capsule. One of the main findings was that the rotator cuff muscles are loaded statically. Moreover, they found that most likely only two simulators had the ability to load the rotator cuff muscles dynamically but did not use the dynamic mode in the presented studies.

In this scoping review, we intend to broaden the search from experimental setups for rotator cuff repair and instability to glenohumeral joint experiments. Specifically, we will describe differences and commonalities of *ex-vivo* glenohumeral experimental set ups and their strengths and limitations.

### Objective and review question

This scoping review seeks to identify methodological and experimental studies that describe or involve glenohumeral joint simulators. The characteristics of these simulators will then be assessed to highlight their strengths and limitations. Particularly, the strengths and limitations will be described by answering the following research questions:

•   What is the state of art of glenohumeral simulators in research where the muscles are explicitly modeled?

•   How accurate are muscle insertion, glenoid fossa and other soft tissues replicated?

•   How are the muscles actuated?

•   How is the system controlled?

## Protocol

### Methods

The proposed scoping review will be conducted in accordance with the Joanna Bricks Institute (JBI) methodology for scoping reviews ^11^. In particular, the search strategy will be pre-defined. This strategy includes search terms, eligibly criteria and how the study selection is performed. The protocol is reported according to the Preferred Reporting Items for Systematic Reviews and Meta-analyses extension for protocols (PRISMA-P) ^12^. The full review will be reported according to the Preferred Reporting Items for Systematic Reviews and Meta-Analyses extension for Scoping Reviews (PRISMA-ScR) ^13^.

### Eligibility criteria

***Inclusion.*** Studies will be included if they are *ex-vivo* (cadaver) or mechanical simulation studies with anatomically accurate artificial humerus and glenoid, and if there are actuated muscle forces of either rotator cuff muscles or deltoid muscle. The motion in the experimental setup has to be induced by the muscle forces (i.e. motions are induced by actuators which mimic the muscle forces, such as cable pulleys) and not through an external guide such as a robotic device. There will be no restriction on language and date of publishing.

***Exclusion.*** All studies of in-vivo or animal nature will be excluded. A study will be excluded if the motion is constrained through something other than anatomical structures. Moreover, if the net forces or moments in the glenohumeral joint are applied externally, the study will be excluded. Passive movements of the humerus are thus generally excluded. Static experiments and tendon extrusion experiments are excluded as well. Lastly, computational musculoskeletal simulation without integrating the results into an *ex-vivo* simulator will be excluded.

### Search strategy

A medical information specialist (HE) drafted the full search for PubMed using text words with synonyms and word variations as well as subject headings around the topic areas *ex-vivo*, simulator, shoulder muscles and biomechanics. These and possible further pertinent terms were discussed in the team. The search was translated using the Polyglot Search Translator ^14^ and internally peer reviewed by another information specialist. The full search strategy ^16^ was used to conduct searches on PubMed, Embase via Elsevier and the Web of Science Core collection.

In addition to the database search, we will conduct backward and forward citation tracking of the included studies using Scopus.

### Search management

All retrieved references will be exported to Endnote 20 (Clarivate Analytics, 2020) and database duplicates removed according to the Bramer method (which includes using customized import/export filters and several rounds of manually changing the deduplication configuration to reduce the risk of false duplicate removal) ^15^. Zotero could also be used to manage the retrieved references. Additional references identified in backward and forward citation tracking will also be managed the same way.

### Study selection/selection of the evidence

Following a pilot test, titles and abstracts will then be screened by two independent reviewers for assessment against the inclusion criteria for the review. Potentially relevant sources will be retrieved in full and their citation details imported into Endnote 20 and retrieved from Endnote and through the University library Basel.

The full text of selected citations will be assessed in detail against the inclusion criteria by two reviewers. Reasons for exclusion of sources of evidence at full text that do not meet the inclusion criteria will be recorded and reported in the scoping review.

Any disagreements that arise between the reviewers at each stage of the selection process will be resolved through discussion, or with an additional reviewer. The results of the search and the study inclusion process will be reported in full in the final scoping review and presented in a PRISMA-ScR flow diagram ^13^.

### Data extraction

The data extracted will include specific details about the ability of the shoulder simulators to simulate physiological conditions and motions. This will be reported in tabular form comprising the data listed below. The data extraction will not be limited to rotator cuff and deltoid muscle but all the glenohumeral muscles that are used in the reviewed studies will be reported. Furthermore, the cadaver preparation will be categorized in fresh frozen/embalming in fluids. Moreover, the condition of the soft tissue will be reported. A draft extraction form is provided (see Table 1). The draft data extraction form will be modified and revised as necessary during the process of extracting data from each included evidence source. Modifications will be detailed in the scoping review. Any disagreements that arise between the reviewers will be resolved through discussion and with an additional reviewer. If appropriate, authors of papers will be contacted to request missing or additional data, where required.

**Table 1.** Data extraction sheet template.

**Scoping review details**			
Scoping review title:	*Ex-vivo* experimental strategies of unconstraint shoulder biomechanics: a scoping review		
Review objective/s:	Review the state of art of unconstraint shoulder biomechanical ex-vivo experiments.		
Review question/s:	What is the state of art of glenohumeral simulators in research, where the muscles are explicitly modeled? How accurate are muscle insertion, glenoid fossa and other soft tissues replicated? How are the muscles actuated? How is the system controlled?		
**Inclusion/exclusion criteria**			
Population	*Ex-vivo*/mechanical simulations with artificial humerus and glenoid		
Concept	Unconstraint glenohumeral joint simulators are included. The muscle forces must be applied actively or at least partially. External guidance and static experiments are the main exclusion criteria.		
Types of evidence source	*Ex-vivo* experimental and mechanical simulation studies		
**Evidence source details and characteristics**			
Citation details (e.g., author/s, date, title, journal, volume, issue, pages)			
Country			
Context			
Participants (details e.g., age/sex and number)			
**Details/results extracted from source of evidence**			
*Mechanical setup*			
DOF and constraints			
Name of the muscles which are represented	active:		passive:
Number of muscles	active:	passive:	total:
Muscle insertion			
Wrapping objects			
Sensors			
Imposed external loads			
*Cadaver preparation*			
Preparation method			
Included tissues			
Condition of the tissues			
*Muscle force estimation*			
Are the muscle force estimation reported?			
How are they estimated?			
*Control concept*			
Is the control strategy reported?			
What is the control structure?			
How are the control gains determined?			

### Quality appraisal

Within the framework of this scoping review, no quality appraisal is planned.

### Data analysis and presentation

This data will be listed in a tabular form and the studies will be ordered from most to least physiological according to available data. If the physiological level is the same, then the studies will be ordered alphabetically.

In addition to the tabular view, we will narratively analyse the results in the review text. Together, these results will provide a comprehensive scope of past research methodologies on this topic and likely identify opportunities on how to further develop such simulators.

### Dissemination of results

The completed review will be published in an open access peer-reviewed journal.

## Study status

Start date of search: June 2021; anticipated completion date of review: February 2022.

Current study status: preliminary searches, yes; piloting of the study selection process, yes; formal screening of search results against eligibility criteria, no; data extraction, no; data analysis, no.

## Data availability

### Underlying data

No underlying data are associated with this article.

### Extended data

Zenodo: *Ex-vivo* experimental strategies for assessing unconstrained shoulder biomechanics: a scoping review's detailed search strategy. https://doi.org/10.5281/zenodo.5734982 ^16^

This project contains the following file:

- DOKU_Search-strat_ *Ex-vivo*-Sim_20210712_hae.pdf: The search strategy of the scoping review, *Ex-vivo* experimental strategies for assessing unconstrained shoulder biomechanics: a scoping review, is elaborated in detail in this document.

Data are available under the terms of the Creative Commons Attribution 4.0 International license (CC-BY 4.0).
