# Supplementary material for: Ex-vivo experimental strategies for assessing unconstrained shoulder biomechanics: a scoping review protocol
Source: F1000Res. 2023 Jan 31;11:77. Originally published 2022 Jan 21. [Version 3] doi: 10.12688/f1000research.72856.3 (PMC9834666; doi:10.12688/f1000research.72856.3)
Supplement: Peer review history

# Peer review: Reviewer response for version 3

**DOI:** 10.5256/f1000research.143543.r161881

**Published:** 2023-04-05

**Authors:** Dirk Maier

**Affiliations:** 1 University of Freiburg, Freiburg, Germany

Approved with no further comments.

Is the study design appropriate for the research question?

Yes

Is the rationale for, and objectives of, the study clearly described?

Yes

Are sufficient details of the methods provided to allow replication by others?

Yes

Are the datasets clearly presented in a useable and accessible format?

Yes

Reviewer Expertise:

Shoulder surgery, shoulder biomechanics, rotator cuff lesions

I confirm that I have read this submission and believe that I have an appropriate level of expertise to confirm that it is of an acceptable scientific standard.

# Peer review: Reviewer response for version 3

**DOI:** 10.5256/f1000research.143543.r161882

**Published:** 2023-01-31

**Authors:** Louis Ferreira, David Axford

**Affiliations:** 1 Department of Mechanical & Materials Engineering, University of Western Ontario, London, ON, Canada

No further comments.

Is the study design appropriate for the research question?

Partly

Is the rationale for, and objectives of, the study clearly described?

Yes

Are sufficient details of the methods provided to allow replication by others?

Yes

Are the datasets clearly presented in a useable and accessible format?

Not applicable

Reviewer Expertise:

In-vitro joint motion simulation; shoulder biomechanics; orthopaedic basic research.

We confirm that we have read this submission and believe that we have an appropriate level of expertise to confirm that it is of an acceptable scientific standard.

# Peer review: Reviewer response for version 2

**DOI:** 10.5256/f1000research.121948.r156345

**Published:** 2023-01-11

**Authors:** Dirk Maier

**Affiliations:** 1 University of Freiburg, Freiburg, Germany

This scoping review aims to benchmark the most advanced glenohumeral simulators. As reviews focusing on unconstrained simulators are scarce, this review provides a good overview of the development of such simulators and their limitations. Thus, it markedly contributes to the improvement of simulators of the next generation. In general, this may be of interest to orthopaedic surgeons and researchers of the shoulder joint, but especially to the biomechanical engineering community, as many aspects of developing and improving such a sophisticated device are being investigated.

 I agree with the authors to choose the design of a scoping review, since the literature provides a wide range of simulators and yields heterogenous data. Furthermore, this scoping review provides a good overview of simulators that avoid the usual simplification of constraining the humerus. Many simulators in the literature constrain the humerus to the abduction plane. This reduces the activation of the stabilizing muscles. On one hand this reduces the complexity to control the humerus, but on the other hand it reduces the importance of the rotator cuff muscles as well.

 The systematic search is well set up and managed by an information specialist and a wide range of synonyms is used which should identify most relevant simulators.

 Current reviews lack the aspect of the control concept, whereas this scoping review aims to identify it. Another strength is that the preparation methods are looked at and how the setup is mechanically designed.

 Although I endorse this scoping review, some aspects should be considered. First, I agree with the previous reviewer that the scapula orientation and motion should be identified, as the joint reaction force varies with the orientation of the glenoid. Second, I also agree with the other reviewer that the robotic involvement should have been closer looked at. Although, I suspect there are only a few or no studies that only include a robot to perturbate the humerus.

 Lastly, this review is a scoping review and not a systematic review but can be justified as there are too few simulators in the literature to be examined in a meta-analysis. Furthermore, the data is too heterogenous to be included in a statistical analysis.

 Overall, the study is thoroughly designed and well described, and relevant previous publications are referenced. In summary, I recommend publication of this scopiong review with some minor revisions.

Is the study design appropriate for the research question?

Yes

Is the rationale for, and objectives of, the study clearly described?

Yes

Are sufficient details of the methods provided to allow replication by others?

Yes

Are the datasets clearly presented in a useable and accessible format?

Yes

Reviewer Expertise:

Shoulder surgery, shoulder biomechanics, rotator cuff lesions

I confirm that I have read this submission and believe that I have an appropriate level of expertise to confirm that it is of an acceptable scientific standard.

# Response

**Published:** 2023-01-23

**Authors:** Jeremy Genter

**Affiliations:** ZHAW, Switzerland

Thank you for highlighting the importance of this scoping review.

 Regarding your suggestion:

 Thank you for pointing out that the scapula orientation should be consider. We have amended this in section: Data extraction, where we elaborated how we plan to extract this data.

 We agree with you that a scoping review is the right choice of methodology.

 Thank you for recommending for publication of our protocol

# Peer review: Reviewer response for version 2

**DOI:** 10.5256/f1000research.121948.r127793

**Published:** 2022-04-19

**Authors:** Louis Ferreira, David Axford

**Affiliations:** 1 Department of Mechanical & Materials Engineering, University of Western Ontario, London, ON, Canada

The authors present the rationale and protocol for a review of current ex-vivo experimental methodologies used to study shoulder biomechanics. The main objectives of this study are to determine the state of art of glenohumeral simulators that explicitly model the muscles of the shoulder. The authors intend to determine the accuracy of tissue replication while highlighting how many muscles each system actuates and how those muscles are actuated. Study selection criteria is outlined, and search methodology is described. The authors provide a template that will be used to extract key data from each study reviewed.

 I commend the authors for performing this scoping review, which I believe will make it easier to place simulators in the landscape and understand where any gaps in the literature may be. The rationale for this study is clearly outlined. The objectives are clearly described and the need for this review is discussed given the limitations of existing literature review studies.

 The authors’ study design is partly appropriate for the research question. Although the study design is largely appropriate for the research question, some adjustments to the data extraction form are recommended.

 Firstly, although a previous review has been conducted that examined scapular orientation, it is still important to consider scapular orientation/motion when reviewing simulators that replicate shoulder motion since scapular motion plays a significant role in shoulder biomechanics. The rhythm between the humerus and scapula is important to shoulder stability because as the arm abducts, the scapula rotates under to provide support against the gravity load vector. Some simulators offer simple uni-planar scapular rotation and others offer more complex six-axis scapular mobility. Therefore, I suggest that scapular motion methods also be included in the review of functionality.

 Secondly, in the “Muscle force estimation” and “Control concept” sections of the form, the questions appear to be tailored towards simulators that operate using force-based control. Although force-based control is the most common method of control, other control schemes exist that may be difficult to categorize based on the questions being asked about the control method. Control methods might be broadly categorized in a four-square in which tendon actuation is either force-based or excursion-based, and the control scheme is either closed-loop or open-loop. There are hybrids to these, but that would cover most. Therefore, I recommend to include some broader questions about the controller design to highlight the differences between control techniques.

 I agree with the authors approach that “a study should be excluded if the motion is constrained through something other than anatomical structures”. Some simulators use a rail or other passive guide to constrain the humerus within a particular elevation plane; however, the authors should be careful that some of these simulators use such a guide device as an adjunct which is removed for some protocols. It will be important not to exclude these simulators entirely, but I agree that if the complete motion pathway is not achieved without the guide then they should be excluded. The authors also state that “passive movements of the humerus such as guidance through a robotic device are thus generally excluded”. This is potentially more difficult to determine. Some simulators are augmented with a robot in order to generate varying external loads during motion, such as forces experienced in activities of daily living. In this scheme, the robot is supposed to be transparent in every other direction – meaning that it should impart zero forces other than the simulated load. In practice, this is very difficult to achieve because it requires near perfect real-time force response. Typically, the robot’s presence adds dampening to the whole system which provides a false stability to the motion controller. I suggest that if such a simulator is reported to produce the full motion without the robot, then it might be included; however, I suspect that this will be a difficult criteria to meet. I agree with the authors’ general strategy that if anything is in contact with the arm, then the first choice should be to exclude the simulator; though sometimes external augments are added for a purpose, so I would still search for other papers of that same simulator to determine whether it does have the ability to run unconstrained or unassisted.

 I see that the search term ‘in-vitro’ is included in the search strategy. This is important, as some groups use that term instead of ex-vivo. The applicability of the term ‘in-vitro’ is debatable but it is still important to give it equal weight in the search because some important simulators are not described by their authors as ex-vivo.

 The study design is clearly described, and relevant papers are referenced to further describe the methodology. There is sufficient detail to allow replication by others.

Is the study design appropriate for the research question?

Partly

Is the rationale for, and objectives of, the study clearly described?

Yes

Are sufficient details of the methods provided to allow replication by others?

Yes

Are the datasets clearly presented in a useable and accessible format?

Not applicable

Reviewer Expertise:

in-vitro joint motion simulation; shoulder biomechanics; orthopaedic basic research.

We confirm that we have read this submission and believe that we have an appropriate level of expertise to confirm that it is of an acceptable scientific standard.

# Response

**Published:** 2023-01-23

**Authors:** Jeremy Genter

**Affiliations:** ZHAW, Switzerland

Thank you for commending us to perform this scoping review.

 Regarding your first adjustment recommendation:

 In the extraction sheet, we will record this under DOF and constraints, although we agree it could be more clearly stated. Therefore, we amended the extraction sheet and elaborated how we plan to extract the data in section: Data extraction.

 Regarding your second adjustment recommendation:

 Thank you for pointing this out. We have amended the extraction sheet and elaborated how we plan to extract the data in section: Data extraction.

 Regarding your suggestion on constraint motion and robotic assistance:

 Thank you for this suggestion. There are indeed some simulators that constrain the motion in their first-generation of the simulator. We will still exclude these studies, but if the same simulator is unconstraint in another study, it then will be included. We agree that if an external force is applied with the sole purpose to perturb or induce an additional external load the study should be included. We have amended this in the exclusion subsection.

 Regarding the search terms:

 We agree that "in vitro" and "ex vivo" should be included, thank you.
